# Effects of Melatonin on Liver of D-Galactose-Induced Aged Mouse Model

**DOI:** 10.3390/cimb45100530

**Published:** 2023-10-17

**Authors:** Ran Lee, Won-Yong Lee, Hyun-Jung Park

**Affiliations:** 1 Department of Livestock, Korea National University of Agriculture and Fisheries, Jeonju 54874, Republic of Korea; ranran2424@gmail.com (R.L.); leewy81@korea.kr (W.-Y.L.); 2 Department of Animal Biotechnology, College of Life Science, Sangji University, Wonju-si 26339, Republic of Korea

**Keywords:** melatonin, hepatic steatosis, D-Galactose, aging model, inflammation

## Abstract

Melatonin, a hormone secreted by the pineal gland of vertebrates, regulates sleep, blood pressure, and circadian and seasonal rhythms, and acts as an antioxidant and anti-inflammatory agent. We investigated the protective effects of melatonin against markers of D-galactose (D-Gal)-induced hepatocellular aging, including liver inflammation, hepatocyte structural damage, and non-alcoholic fatty liver. Mice were divided into four groups: phosphate-buffered saline (PBS, control), D-Gal (200 mg/kg/day), melatonin (20 mg/kg), and D-Gal (200 mg/kg) and melatonin (20 mg) cotreatment. The treatments were administered once daily for eight consecutive weeks. Melatonin treatment alleviated D-Gal-induced hepatocyte impairment. The AST level was significantly increased in the D-Gal-treated groups compared to that in the control group, while the ALT level was decreased compared to the melatonin and D-Gal cotreated group. Inflammatory genes, such as IL1-β, NF-κB, IL-6, TNFα, and iNOS, were significantly increased in the D-Gal aging model, whereas the expression levels of these genes were low in the D-Gal and melatonin cotreated group. Interestingly, the expression levels of hepatic steatosis-related genes, such as LXRα, C/EBPα, PPARα, ACC, ACOX1, and CPT-1, were markedly decreased in the D-Gal and melatonin cotreated group. These results suggest that melatonin suppresses hepatic steatosis and inflammation in a mouse model of D-Gal-induced aging.

## 1. Introduction

Aging is a universal, degenerative, and complex process in which damage accumulates in the body as a biological response to environmental, dietary, and genetic factors, resulting in progressive and irreversible functional decline in all organ systems [1]. Previous studies have repeatedly reported that aging increases the incidence of geriatric diseases, including diabetes [2], Alzheimer’s disease [3], Parkinson’s disease [4], cardiovascular disease [5], and chronic obstructive pulmonary disease [6]. The rate of aging is related to an increase in oxidative stress, which modulates the efficiency of regulatory systems, such as the immune, nervous, and endocrine systems [7]. In the liver, which is involved in homeostasis and immunity, aging can induce hepatic steatosis, hepatocyte necrosis, and liver failure [8]. In particular, the restriction of the aging process in hepatocytes has been reported in previous studies of antioxidants and inflammation [9]. D-Galactose (D-Gal) is a monosaccharide that is abundant in dairy products and fruits. The recommended D-Gal intake during growth in humans is 0.5–3.5 g/kg/day, and it is metabolized and excreted within 8 h of ingestion [10]. Consuming low concentrations of D-Gal daily helps to improve health during early human development [10,11]. Intakes of 0.5–1 g/kg/day of D-Gal are excreted within 8 h after metabolism, are safe for patients, and improve physical function [10]. For example, when a low concentration of D-Gal is dissolved in drinking water and fed to mice and rats, approximately 88% is absorbed in the gastrointestinal tract and has a buffering capacity for liver retention [12]. However, exposure to excessive concentrations of D-Gal accelerates overall aging-related changes. Xu reported the first study that showed accelerated aging in Kunming mice in response to treatment with D-Gal for 8 weeks, and this approach has been used to induce aging [13]. The dose of D-Gal for inducing liver aging is administered intraperitoneally or subcutaneously for 6–12 weeks in the range of 50–1200 mg/kg/day [14]. D-Gal-induced aged mice show symptoms similar to those of non-alcoholic fatty liver disease (NAFLD) and non-alcoholic steatohepatitis (NASH), which are caused by the production of ROS [15,16]. Overexposure to D-Gal not only increases free radical production but also disrupts the immune response. Inflammation is one of the main causes of the processes that are closely linked to oxidative stress that lead to pathological changes [17]. Metabolic diseases associated with aging present major health problems, and their prevalence and incidence continue to increase independently of the study period. The prevention and treatment of diseases caused by aging are still lacking, despite advances in medical technology. A relationship between liver damage and oxidative stress has been well established [18]. Experimental models of D-Gal-induced liver damage have provided evidence that the morphological and functional characteristics of human hepatitis are almost identical [19,20,21]. Previous studies have suggested that inflammation is a major cause of liver damage [9,22].

Melatonin (N-acetyl-5-methoxytryptamine) is primarily synthesized from tryptophan in mammalian pineal glands [23]. In animals, melatonin maintains circadian homeostasis, enhances immunity, and regulates sleep and the reproductive system via multiple receptors. In addition, melatonin improves stress resistance, reduces oxidative stress, and promotes seed germination in plants [24]. Endogenous melatonin, which can be consumed in food, is abundant in berries and nuts and has been studied as an antioxidant [25]. Recent studies have focused on the role of melatonin in scavenging free oxygen radicals, protecting cells and tissues from radical-induced damage, and promoting lipid metabolism. Numerous studies have demonstrated the beneficial effects of melatonin on liver damage and disease [26]. Nevertheless, the role of melatonin in the livers of D-Gal-induced aging mice has not yet been investigated. In this study, we used D-Gal to induce senescence in mice and investigated whether melatonin could reduce liver damage.

## 2. Materials and Methods

### 2.1. Animals and Treatments

Six-week-old male *CD-1* mice were obtained from the Animal Centre of Dae Han Bio Link Co. (Daejeon, Republic of Korea). The mice were housed under constant conditions (humidity 40–60%, 12 h light and dark cycle, temperature 20–25 °C). After 7 d of acclimatization, the experimental animals were randomly divided into four groups of six mice each, as follows: control, D-Gal treatment (200 mg/kg/day), melatonin (20 mg/kg/day), and D-Gal (200 mg/kg/day) and melatonin (20 mg/kg/day) cotreatment. The D-Gal (Sigma Aldrich, St. Louis, MO, USA) was dissolved in normal saline; the melatonin (Sigma Aldrich) was first dissolved in 0.1% DMSO (Sigma Aldrich) and then in saline to reach the final administered volume. The control and treatment groups were injected intraperitoneally once daily for 8 weeks. All experimental animals were weighed weekly. At the end of the experiment, the mice were euthanized, and their blood and livers, cleaned with saline, were collected. This animal study was performed in accordance with the guidelines established by the Institutional Animal Care and Use Committee of Sangji University, who approved the experimental protocol (No. 2021-20).

### 2.2. Liver Index Determination

Blood and liver samples were collected from the experimental mice 30 min after the completion of the experiment, and the liver index was determined using the following Equation:

$$\mathrm{Liver}\;\mathrm{index}\;(\%)=100\times \frac{Weight\;of\;liver\;(g)}{Body\;weight\;(g)}$$

### 2.3. Cell Culture and Treatment

The human hepatoma cell line HepG2 was obtained from the Korean Cell Line Bank (KCLB, Seoul, Republic of Korea) and cultured in DMEM (Gibco, Carlsbad, CA, USA) with 10% FBS (Gibco, Carlsbad, CA, USA) and 1% Penicillin–Streptomycin (10,000 U/mL, Gibco, Carlsbad, CA, USA) in a humidified atmosphere of 5% CO_2_ at 37 °C. Lipopolysaccharide (LPS, Sigma Aldrich) and melatonin stock solutions were dissolved in DPBS (Gibco, Carlsbad, CA, USA) to 1 M solution and then diluted to the desired concentration for the experiment.

### 2.4. Histological Analyses

Histological analyses of the liver tissue and immunostaining were performed as previously described [27,28]. Liver samples were fixed in a 10% neutral buffered formalin solution overnight at 4 °C and then gradually dehydrated using 30–100% ethanol for 120 min. The dehydrated tissue was incubated with xylene for 120 min at RT in a clearing step, embedded in paraffin blocks, and these were cut into 4 µm thick sections using a microtome (Leica, Nussloch, Germany). The sectioned samples were stained with hematoxylin and eosin (H&E), and images were obtained using a microscope.

### 2.5. Immunofluorescence

The liver slides were deparaffinized in xylene for 10 min and rehydrated with 100, 95, 90, 80, and 70% H_2_O for 5 min each. For antigen unmasking, citrated buffer (0.01 M) was added, and the mixture was boiled for 15 min. HepG2 cells were fixed in 10% neutral-buffered formalin solution for 10 min at RT and washed thrice with DPBS. The immunofluorescence primary antibodies are listed in Table 1. The first antibodies were purchased from Bio-Rad (Hercules, CA, USA) and Santa Cruz Biotech (Dallas, TX, USA). The secondary antibodies were as follows: Alexa Fluor^®^ 488 goat anti-mouse IgG, Alexa Fluor^®^ 488 donkey anti-rabbit IgG (Jackson ImmunoResearch Laboratories, West Grove, PA, USA), Alexa Fluor^®^ 488 donkey anti-goat IgG, and Alexa Fluor^®^ 568 donkey anti-mouse IgG (Life Technologies, Carlsbad, CA, USA). Images were obtained using a fluorescence microscope (Olympus IX73; Tokyo, Japan) equipped with an eXcope camera.

### 2.6. Western Blotting

Total proteins from the liver tissue and HepG2 cells were extracted using RIPA buffer (Thermo Fisher Scientific, Waltham, MA, USA) containing a protease inhibitor cocktail (Roche, Mannheim, Germany), and were carefully homogenized at 4 °C. Tissue or cell debris was removed via centrifugation and the supernatant was collected. The protein concentrations were determined using the BCA method [29]. and the protein samples (30 μg/lane) were loaded and separated on 10% acrylamide gel and 4–20% mini TGX gels (Bio-Rad, Hercules, CA, USA; #456–1096) [29]. They were transferred onto polyvinylidene difluoride membranes, incubated overnight with primary antibodies (dilution, 1:2000 in Tween 20/Tris-buffered saline containing 1% bovine serum albumin) at 4 °C. The primary antibodies are listed in Table 1. After washing thrice with TBST, the samples were incubated with anti-mouse or anti-rabbit horseradish peroxidase-conjugated secondary antibodies (Santa Cruz, Dallas, TX, USA) for 60 min at 24 °C. The protein signals were visualized using an ECL substrate (Thermo Scientific, Rockford, IL, USA). β-actin was used as the control for normalization.

### 2.7. RNA Isolation and Quantitative PCR

Total RNA was isolated from the liver tissue and HepG2 cells using a Qiagen RNeasy Mini Kit (Qiagen, Redwood, CA, USA) with on-column DNase treatment (Qiagen), according to the manufacturer’s instructions, and then reverse-transcribed for cDNA synthesis using a RevertAid First Strand cDNA synthesis kit (Thermo Fisher Scientific). A QuantStudio 1 instrument (Applied Biosystems, Foster City, CA, USA) was used for quantitative real-time PCR (qPCR) with a SYBR mixture (Bioneer, Daejeon, Republic of Korea). The PCR reaction was performed at 94 °C for 1 min, followed by 40 cycles at 94 °C for 10 s, 57 °C for 10 s, and 72 °C for 20 s. The expression levels were calculated from the cycle threshold (Ct) value using the ΔCt method of quantification (2^−ΔΔCt^ method), normalized to endogenous gene levels. The primers used are listed in Table 2.

### 2.8. Detection of IL1-β, AST, and ALT Levels

Mouse serum was clarified via centrifugation and stored at −80 °C. The mouse IL-1β levels in the supernatants were quantified using uncoated ELISA Kits (Thermo Fisher Scientific, Waltham, MA, USA, cat; #88-7013-88) following the manufacturer’s instructions [30]. The absorbance was read on a BioTek Epoch Microplate reader (Winooski, VT, USA) at a 450 nm wavelength. The ALT and AST levels in the mouse serum were measured by Samkwang Medical Laboratories (Seoul, Republic of Korea).

### 2.9. Statistical Analysis

All data are expressed as group means ± standard error, and all experiments were performed independently at least thrice. Data analysis was performed using the SPSS statistical package ver. 25.0 for Windows (IBM Corp., Armonk, NY, USA). Comparisons among the groups were evaluated using a one-way ANOVA, followed by Tukey’s honest significant difference test. The significance was set at *p* < 0.05 and *p <* 0.01. Graphs were generated using Sigma Plot 8.0.

## 3. Results

### 3.1. Melatonin Protected Mice against D-Gal-Induced Liver Dysfunction

**Figure 1:**
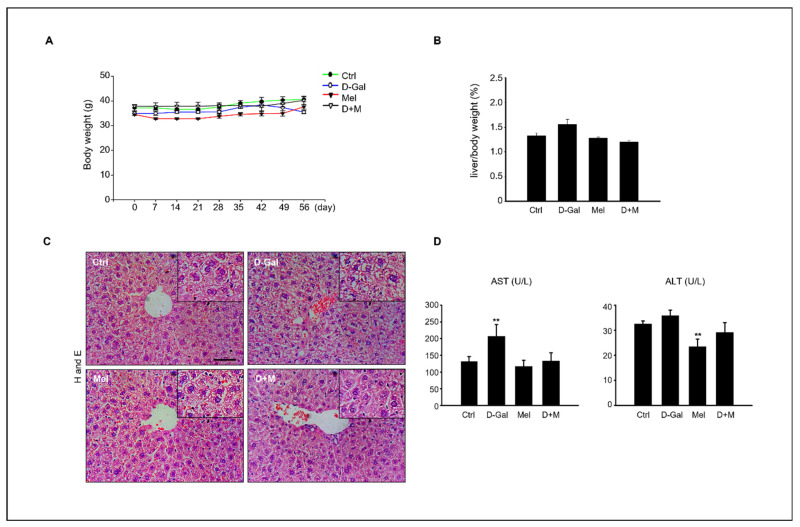
Effect of melatonin on D-Gal-induced liver damage. (**A**) Body weight variation in control, D-Gal, melatonin, and D-Gal with melatonin groups (*n* = 6 in each) during the experiment. (**B**) Relative liver index (%) of control, D-Gal, melatonin, and D-Gal with melatonin groups. (**C**) Histological assessments in liver tissue of D-Gal, melatonin, and D-Gal with melatonin groups. Scale bar = 500 µm. (**D**) Serum AST and ALT levels in each experiment group. The data are shown as mean ± SE with four-fold determinations per condition. ** *p* < 0.01.

Compared with the control group, the D-Gal group showed a decrease in body weight (g) and an increase in liver index (%), but the difference was not significant (*p* < 0.05, Figure 1A,B). The liver structures of mice in the control and melatonin groups exhibited normal polygonal-shaped hepatocytes, whereas those in the D-Gal-treated groups exhibited liver damage, including dilated and ballooned hepatocytes, which are found particularly in steatohepatitis. However, both the melatonin-treated and cotreated groups showed remarkably suppressed morphological change (Figure 1C). In addition, the administration of D-Gal significantly increased the levels of aspartate aminotransferase (AST) compared to those in the other groups, although the alanine aminotransferase (ALT) levels did not differ between the control and D-Gal-treated groups. This result indicated that melatonin suppressed liver morphological changes and increased AST levels following D-Gal treatment in mice (Figure 1D).

### 3.2. Melatonin Ameliorated the Hepatic Lipid Profiles in D-Gal-Induced Liver Disease in Aging Mice

**Figure 2:**
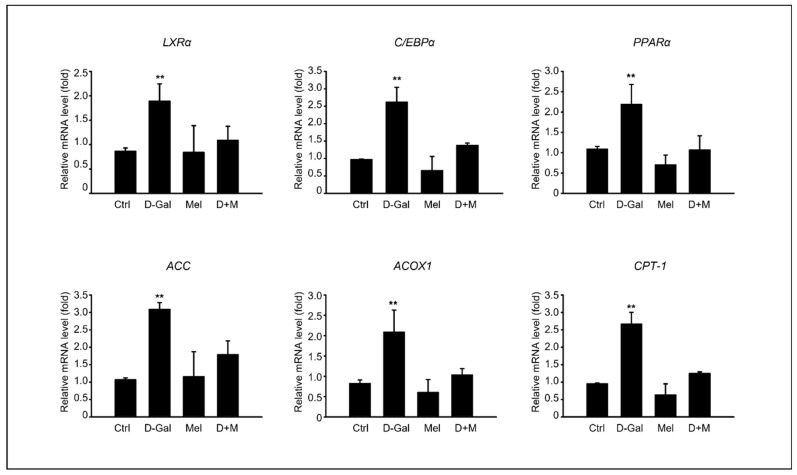
Melatonin inhibits hepatic lipid accumulation in D-Gal-induced liver disease. mRNA expression of fatty acid β-oxidation-related gene expression, including CPT1, ACOX1, LXRα, ACC, C/EBPα, and PPARα. The data are shown as mean ± SE with four-fold determinations per condition. ** p < 0.01.

To investigate the mechanisms by which melatonin suppresses D-Gal-induced liver damage, we determined whether the inflammatory and hepatic steatosis responses of the liver were induced by D-Gal treatment and confirmed whether melatonin could inhibit the inflammatory response in the D-Gal-treated groups. The fatty acid β-oxidation-related gene expression, including LXRα, C/EBPα, PPARα, ACC, ACOX1, and CPT-1, were evaluated. The mRNA levels of transcription factors, such as *LXRα* and *C/EBPα,* were significantly higher in D-Gal group compared to those in the control group and the D-Gal groups supplemented with melatonin. Similarly, we found upregulation patterns in energy metabolism-related genes, including *PPARa*, *ACC*, *ACOX1*, and *CPT1* (Figure 2). Interestingly, the melatonin and D-Gal with melatonin groups showed a decreased expression of these genes compared to the D-Gal group. These results indicated that melatonin ameliorated the hepatic lipid levels in D-Gal-induced hepatic steatosis.

### 3.3. Melatonin Suppressed Pro-Inflammatory Cytokines in Induced Liver Damage in Aging Mice

**Figure 3:**
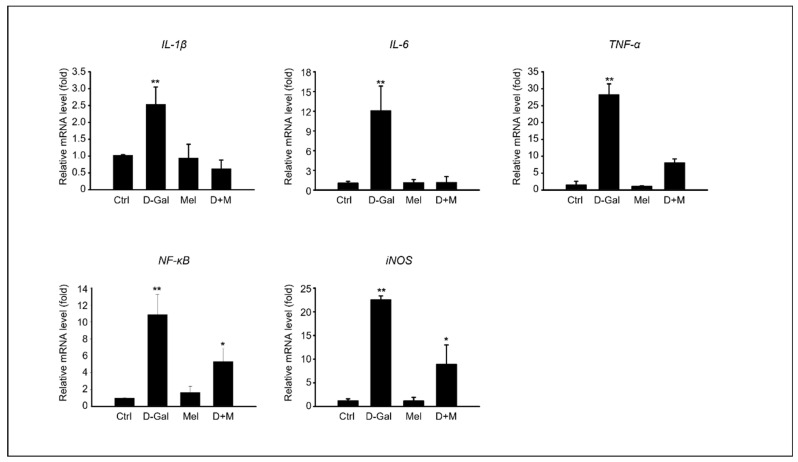
Melatonin reduces pro-inflammatory cytokines in induced liver damage in aging mice. mRNA expression of inflammation-related genes, such as *IL-1β*, *IL-6*, *TNF-α*, *NF-kB*, and *iNOS.* The data are shown as mean ± SE with four-fold determinations per condition. * p < 0.05 and ** p < 0.01.

**Figure 4:**
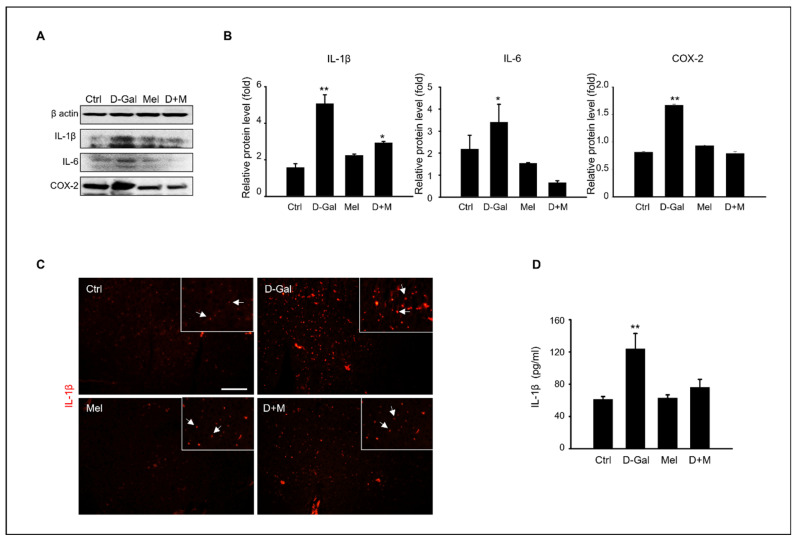
Melatonin modulation of pro-inflammatory cytokines in induced liver damage in aging mice. (**A**) The protein level of inflammation-related expression, such as IL-1β, IL-6, and COX-2. (**B**) The relative inflammation protein/β-actin level. (**C**) Analysis of IL-1β expression in liver tissue of control, D-Gal, melatonin, and D-Gal with melatonin group via immunofluorescence. The white arrows indicated that enlarged IL-1b expression cells in liver tissue. Scale bar = 50 µm. (**D**) Analysis of IL-1β level of serum in control, D-Gal, and D-Gal with melatonin groups. The data are shown as mean ± SE with four-fold determinations per condition. * *p* < 0.05 and ** *p* < 0.01.

The mRNA level of inflammation-related genes, including *IL-1β*, *IL-6*, and *TNF-α*, significantly increased under D-Gal treatment, whereas the mRNA level of the melatonin and D-Gal with melatonin groups significantly decreased. Additionally, the mRNA levels of *iNOS* in cells stimulated by cellular cytokines increased in the D-Gal treatment group and significantly decreased in the melatonin and D-Gal treatment groups (Figure 3). As expected, the expression of protein levels related to the inflammatory response, namely IL-1β, IL-6, and COX-2, were higher in the D-Gal groups than in others (Figure 4A,B). Additionally, immunofluorescence and enzyme-linked immunosorbent assays (ELISAs) were performed to verify the gene and protein expression results. IL-1B-stained cells were more reduced in the liver tissue of the D-Gal with melatonin group than in that of the D-Gal group (Figure 4C). In addition, the ELISA was used to confirm that the level of IL-1β in mouse serum in the D-Gal group decreased when melatonin was added (Figure 4D). We demonstrated that melatonin is an inflammatory modulator that mediates the production of cytokines activated by D-Gal.

### 3.4. Melatonin-Enhanced Stabilization of LPS-Induced Inflammatory Mediators in HepG2 Cells

**Figure 5:**
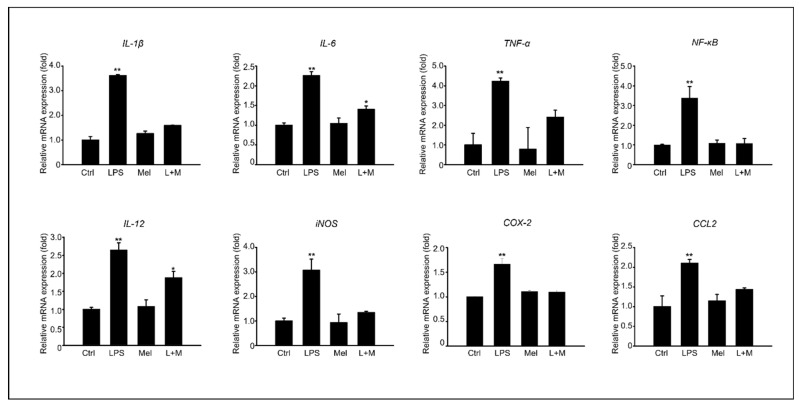
Melatonin improves stabilization of LPS-induced inflammatory mediators in HepG2 cells; of mRNA expression of inflammation-related genes, such as *IL-1β*, *IL-6*, *TNF-α*, *NF-κB*, *IL-12*, *iNOS*, *COX-2*, and *CCL2* in HepG2 cells. The data are shown as means ± SE with four-fold determinations per condition. * p < 0.05, ** p < 0.01.

After confirming the in vivo anti-inflammatory effect of melatonin in a D-Gal aging mouse model, we examined the anti-inflammatory effect of chemokine-related gene expression in LPS-induced cells. HepG2 cells were treated with 100 µM of melatonin in the presence or absence of 1 ug/mL of LPS. Figure 5 shows the results of analyzing the gene expression levels of pro-inflammatory cytokines at the mRNA level in HepG. Compared to the control group, LPS stimulated a substantial increase in the mRNA level of the inflammatory cytokines *IL-1β*, *IL-6*, and *TNF-α* in HepG2 cells. The addition of melatonin significantly decreased *IL-1β*, *IL-6*, and *TNF-α* mRNA levels after LPS stimulation. Melatonin also reduced the mRNA levels of *iNOS* and *COX-2*, key enzymes involved in fatty acid metabolism during LPS-induced inflammation. Furthermore, melatonin markedly inhibited LPS-induced *NF-κB* activation. We confirmed that inflammatory cytokines and enzymes were increased in HepG2 cells by LPS at the gene expression level, and the addition of melatonin to cells improved the stabilization of inflammatory mediators compared to the control and LPS treatment.

### 3.5. Melatonin-Regulated Stabilization of LPS-Induced Inflammatory Mediators in HepG2 Cells

**Figure 6:**
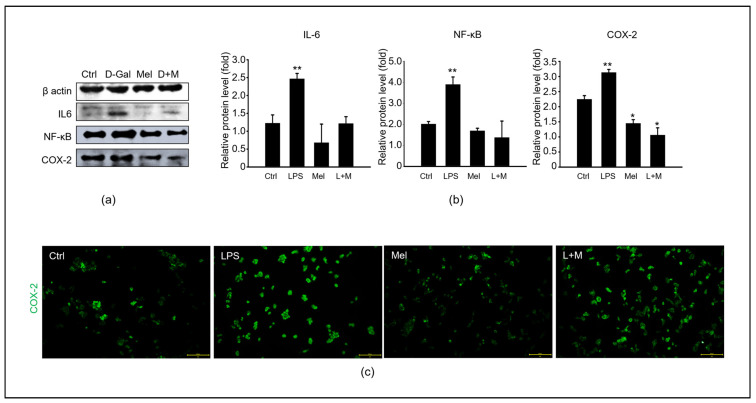
Melatonin improves the stabilization of LPS-induced inflammatory mediators in HepG2 cells. (**a**) The protein levels of an inflammation-related expression, including IL-6, NF-κB, and COX-2. (**b**) The relative inflammatory protein/β-actin levels. (**c**) Analysis of COX-2 expression in HepG2 cells of control, LPS, melatonin, and LPS with melatonin groups via immunofluorescence. Scale bar = 200 µm. The data are shown as means ± SE with four-fold determinations per condition. * p < 0.05, ** p < 0.01.

To evaluate whether melatonin could modulate inflammation in vitro, we added melatonin to LPS-treated HepG2 cells at the protein level (Figure 6). Compared to the control group, LPS stimulated a substantial increase in the protein level of the inflammatory cytokines IL-6, NF-κB, and COX-2 in HepG2 cells. Immunocytochemistry confirmed that melatonin decreased the LPS-induced protein expression level of COX-2. Overall, melatonin simultaneously reduced the mRNA and protein levels of oxidation-induced inflammation in HepG2 cells.

## 4. Discussion

We used a mouse model of D-Gal-induced aging to clarify the role of melatonin in D-Gal-induced liver dysfunction. Fatty liver occurs when fat is not properly metabolized and can be caused by the accumulation of monosaccharides in the body [31,32]. Non-alcoholic fatty liver refers to a wide spectrum of liver diseases that can be caused by simple fatty liver with inflammation, chronic non-alcoholic steatohepatitis, and liver fibrosis through liver cirrhosis as the disease progresses [33]. Aging involves complex changes in biological functions, including changes in glucose, lipid, amino acid, and nucleotide metabolism. Aging and metabolic abnormalities are associated with NAFLD progression in various predictive models [34]. In addition, the age-related decline in the liver metabolic capacity has been shown to increase vulnerability to acute liver injury and susceptibility to fibrotic responses, which is a reason for the increased severity of liver diseases, including hepatitis [35].

The present study confirmed the elimination of D-Gal-induced hepatic hypofunction, inflammation, and steatosis in a mouse model of D-Gal-induced aging. The main indicators for evaluating liver health are the liver index, serum ALT and AST concentrations, and histological pathology [36,37,38]. According to our results (Figure 1), a comparison of liver enzymes in mouse serum showed that the AST concentration was significantly increased in the D-Gal group compared to that in the control group, whereas melatonin decreased the ALT. In addition, cotreatment with D-Gal and melatonin decreased the AST and ALT levels. A histological analysis showed that the hepatocytes around the central vein were enlarged in the control group, whereas the abnormally polygonal swollen hepatocytes were restored in the D-Gal and melatonin cotreatment group. The concentrations of D-Gal that increased the ALT and AST levels in mouse blood have previously been described as follows: BL/6 J mice: 1200 mg/kg/day [39]; Razi mice: 500 mg/kg/day [40]; C57BL/6 mice: 800 mg/kg/day [41]; and ICR: 150 mg/kg/day [42], which supports our results of decreased liver enzyme levels with D-Gal administration. The improvement in liver health indices with supplemental administrations of melatonin suggests a significant protective effect against D-Gal-induced liver damage. Melatonin has been documented to exhibit anti-inflammatory, antioxidant, anticarcinogenic, and circadian rhythm regulatory effects [43,44,45]. Fatty acid synthesis is a complex reaction that occurs in the hepatocyte cytoplasm [46]. Fatty acid synthesis is influenced by the transport activity of mitochondrial citrate transporter (*CIC*) [47] and acetyl-CoA carboxylase (*ACC*). The induction of adipogenic genes was assessed with sterol regulatory element binding proteins (*SREBPs*), carbohydrate response element binding proteins (*ChREBPs*), liver X receptor α (*LXRα*), and peroxisome proliferator-activated receptors (*PPARs*) [48]. The lipid accumulation-related mRNA levels increased in the D-Gal-treated mouse group compared to those in the control and melatonin groups, whereas these mRNA levels decreased in the D-Gal and melatonin cotreated group. Peet et al. reported that LXRα-null mice who were fed a high-cholesterol diet showed a high cholesterol accumulation in the liver, but wild-type mice showed a marked resistance to cholesterol accumulation [49]. PPARs are divided into α, β, and δ types, and regulate lipid homeostasis in various organs [49]. Among them, PPARα regulates lipid metabolism in the liver and is related to hepatic steatosis, steatohepatitis, steatofibrosis, and liver cancer [50]. Melatonin (10, 20, or 50 mg/kg/day) was administered to hamsters with HFD-induced hyperlipidemia; the melatonin treatment reduced the activity of ACC lipogenic enzymes and decreased the mRNA levels of CPT-1 lipolytic enzymes [51]. Liu et al. also reported that injections of 10 mg/kg/day of melatonin in guinea pigs with HFD-induced glucose and lipid metabolism disorders activated the CPT-1 pathway in the liver [52]. ACC stimulates malonyl-CoA synthesis, the rate-limiting step in fatty acid biosynthesis, whereas FAS catalyzes the final step in fatty acid synthesis. Chen et al. showed that the upregulation of *SREBP-1c* and *ACC* mRNA levels induced by lipopolysaccharide (LPS) in hamster livers was attenuated after melatonin treatment [53]. Unexpectedly, the relative mRNA expression of *CPT-1* was upregulated after D-Gal treatment, which was inconsistent with our findings. Although some studies have reported that a melatonin dose of 20 mg/kg significantly increased *CPT-I* mRNA expression, Heo et al. found that *CPT-I* levels did not change significantly after HFD or melatonin administration [54]. This correlation is expected to be dependent on the melatonin concentration, which induces hepatic steatosis. We speculate that the effect of melatonin on the reduction of hepatic fatty acid production at the mRNA expression levels of *PPAR* and CPT-1 is not consistent with previous studies for the following reasons: Compared with HFD-induced obese animal models, D-Gal-induced mice show less weight gain and changes in the liver index, suggesting that the effect of melatonin on regulating fatty acid oxidation pathways may not be significant. This explanation is supported by changes in the liver index, which were not statistically different between the groups. Consistent with previous findings, the mRNA levels of *LXRα*, *C/EBPα*, *ACC*, and *ACOX1* downregulated in our result, suggesting that melatonin may modulate hepatic lipid metabolism. Eventually, hepatic steatosis directly contributes to liver injury by affecting cellular organelles, such as the endoplasmic reticulum and mitochondria, via lipid accumulation in hepatocytes. This results in impaired fatty acid metabolism and excessive free fatty acid metabolism. These effects can lead to apoptosis, necrosis, and inflammation [55]. iNOS and COX-2 are pro-inflammatory factors that regulate the acute-phase response, leading to fibrosis, apoptosis, and damage to cells and organs [56]. The levels of these inflammatory cytokines were significantly decreased in the D-Gal and melatonin cotreated group compared to the D-Gal-injected group, suggesting that melatonin may have potent anti-inflammatory activity in the D-Gal-induced aging mice [26,57]. The senescence-associated secretory phenotypes include IL-6 and TNF-α, which are released to respond and protect host cells from inflammation, damage, irritation, and disease, while indicating chronic inflammation. These cytokines are usually stimulated by the activation of inflammation [58,59,60]. In our results, the expression of *IL-6, NF-κB*, and *iNOS* of mRNA as well as the protein levels were significantly increased in the liver of D-Gal-treated mice and the LPS-treated HepG2 cells. TNF-α secretion is regulated by the NF-κB signaling pathway in the liver, and NF-κB was reported to regulate inflammation [61,62]. Escribano et al. reported that melatonin controls the translocation of NF-κB to the nucleus as well as binding to DNA, preventing rapid increases in the transcription and translation of inflammatory cytokines, including IL-1β, IL-6, and TNF [63]. This study supports our results regarding the role of melatonin as an anti-inflammatory modulator in the liver. Crespo et al. showed that melatonin inhibited NF-κB activation in LPS-stimulated macrophages [57]. We analyzed the expression of IL-6 and TNFα, as well as inflammation-related biomarkers, such as IL-1β, IL-12, and NF-κB. D-Gal and LPS increased the mRNA levels of the reactive oxygen species COX-2, which was attenuated by melatonin. Previous studies have shown that melatonin protects against oxidative stress and reduces the production of inflammatory factors [64]. Previous studies have suggested that NAFLD is associated with epigenetic processes, including DNA methylation, histone modification, and non-coding RNA [65]. DNA methylation is a fundamental epigenetic modification of DNA that occurs at cytosine bases within cytosine-phosphoguanine (CpG) dinucleotides [66,67]. A diet that is depleted of methyl donors reduces hepatic SAM levels and induces the CpG island demethylation of 164 genes that are involved in DNA damage/repair, lipid and glucose metabolism, and fibrogenesis in the mouse liver [68]. Methyl donor feeding, containing folic acid, betaine, choline, and vitamin B12, is involved in the development of obesity and lipid metabolism in association with sterol regulatory element-binding protein 2 (Srebf2) and 1-acylglycerol-3-phosphate O, which modify mRNA expression, and positively affects DNA methylation patterns in the promoter regions of acyltransferase 3 (Agpat3) and estrogen receptor 1 (Esr1) genes, reversing liver fat accumulation in mice that are fed a diet high in fat and sugar [69]. Additionally, methyl donor administration improved HFS-induced NAFLD through fatty acid synthase (Fasn) DNA hypermethylation [70]. In particular, the offspring of HFD-exposed mice exhibit postnatal liver dysfunction due to inherited DNA methylation [71,72]. In the offspring of HFD-fed dams, the cell cycle inhibitor, cyclin-dependent kinase inhibitor 1A (Cdkn1a), is demethylated, ultimately inhibiting hepatocyte growth [73]. Interestingly, it has been reported that the expression of Cdkn1a, which is decreased by radiation, is upregulated in the peripheral blood of rats [74]. Furthermore, melatonin reverses leptin methylation and expression, which are closely associated with hepatic steatosis, and reduces inflammation and chronic hepatic steatosis without apoptosis or histone deacetylation in prenatally dexamethasone-exposed livers with steatosis in young rats [75]. Therefore, based on our findings, melatonin can be used as an anti-inflammatory and anti-steatosis agent in the liver [76,77]. Here, we demonstrated that melatonin regulates cellular and hepatic metabolism and inflammation, and that interactions between these biological processes are associated with increased hepatocellular damage and impaired liver function. In particular, the increased liver inflammation observed in mouse models is exacerbated by the D-Gal-induced aging burden and the increased expression of genes associated with NAFLD. In summary, our findings suggest that melatonin has a positive effect on liver metabolism, inflammatory aging, and non-alcoholic fatty liver.

## Figures and Tables

**Table 1:** List of antibodies used for immunostaining and Western blotting.

Antibody	Company	Catalog Number	Dilution
NF-κB	Santa Cruz Biotech	sc8008	1:2000
IL-6	Bio-RAD	AHP424	1:500
IL-1B	Bio-RAD	MCA1658	1:3000
COX-2	Santa Cruz Biotech	sc376861	1:2000
β-actin	Santa Cruz Biotech	sc47778	1:2000

**Table 2:** Primers designed for qPCR using mouse and human cDNA.

Species	Gene	Forward Primer	Reverse Primer
Mouse	*IL-1B*	5′-ACCTTCCAGGATGAGGACATGA-3′	5′-CTAATGGGAACGTCACACACCA-3′
	*NF-κB*	5′-TGGAGTTCGTGACCGCCGCCG-3′	5′-GCTGGCTCTGCCGGGAAGATG-3′
	*IL-6*	5′-TGATGCTGGTGACAACCACG-3	5′-CAGAATTGCCATTGCACAACTC-3′
	*TNF-α*	5′-CAGGCGGTGCCTATGTCTC-3′	5′-CGATCACCCCGAAGTTCAGTAG-3′
	*iNOS*	5′-GTTCTCAGCCCAACAATACAAGA-3′	5′-GTGGACGGGTCGATGTCAC-3′
	*Gapdh*	5′-GTCGGTGTGAACGGATTTG-3′	5′-CTTGCCGTGGGTAGAGTCAT-3′
Human	*IL-1B*	5′- GTCCTGCGTGTTGAAAGATG-3′	5′-CTGCTTGAGAGGTGCTGATG-3′
	*TNF-α*	5′-GGCGTGGAGCTGAGAGATAAC-3′	5′-TGATGGCAGAGAGGAGGTTG-3′
	*IL-6*	5′-CCAAGGTCGGCTACTGAAAG-3′	5′-GATCCATAGCTGCGTGTCTTC-3′
	*iNOS*	5′-AACATCAGGTCGGCCATTAC-3′	5′-ACTGGGTGAACTCCAAGGTG-3′
	*IL-12*	5′-CCTCCTCCTTGTGGCTACC-3′	5′-GAGTTTGTCTGGCCTTCTGG-3′
	*CCL2*	5′-TCTGTGCCTGCTGCTCATAG-3′	5′-GAGTTTGTCTGGCCTTCTGG-3′
	*NF-k* *B*	5′-TCTGTGTTTGTCCAGCTTCG-3′	5′-AGCTCCAGCACCACTACCAC-3′
	*Gapdh*	5′-GGCTCTCCAGAACATCATCC-3′	5′-CCTGCTTCACCACCTTCTTG-3′
	*COX-2*	5′-TGAGCATCTACGGTTTGCTG -3′	5′-TGCTTGTCTGGAACAACTGC-3′

## Data Availability

The datasets generated and/or analyzed in the current study are available from the corresponding authors upon reasonable request.
